# Implant geometry as a patient-specific identifier in breast brachytherapy: leveraging electromagnetic tracking to prevent treatment mix-ups

**DOI:** 10.1016/j.tipsro.2026.100415

**Published:** 2026-05-22

**Authors:** Christopher Dürrbeck, Vratislav Strnad, Christoph Bert

**Affiliations:** a Department of Radiation Oncology, Universitätsklinikum Erlangen, Friedrich-Alexander-Universität Erlangen-Nürnberg, Erlangen, Germany; b Comprehensive Cancer Center, Universitätsklinikum Erlangen, Erlangen, Germany

**Keywords:** Patient identification, Treatment error, Electromagnetic tracking, Interstitial brachytherapy, Quality assurance, Error detection, Breast cancer

## Abstract

**Background and purpose:**

Patient or treatment plan mix-ups are among the most serious patient-specific human errors in brachytherapy. However, many brachytherapy departments rely only on review by a second, independent person, which does not eliminate the risk of human failure. In this work, we developed and retrospectively evaluated an automated patient identification method based solely on the geometry of the interstitial implant in breast cancer patients.

**Materials and methods:**

The implant geometry is assessed using an electromagnetic tracking (EMT) system that provides real-time positional data of each catheter with sub-millimetre accuracy. The measured implant geometry is rigidly registered to the CT-based implant geometry associated with the clinical treatment plan. To quantitatively compare them, a similarity metric based on a distance-to-agreement (DTA) criterion (3–10 mm) and a pass rate threshold (50–95%) was used. The implants of 80 patients were included in the evaluation, resulting in 6400 patient-treatment plan combinations.

**Results:**

The classifier reliably identified patients with an area under the receiver operating characteristic (ROC) curve close to 1, highlighting an overall excellent discriminative performance. At the optimal decision threshold under the requirement of a false positive rate of 0%, it achieved a sensitivity between 94.8% and 97.5% depending on the DTA and pass rate thresholds, and an overall accuracy of 99.9%.

**Conclusion:**

Interstitial implants in breast brachytherapy are virtually unique, so determining their geometry prior to each fraction is a viable option for patient identification. The EMT-based automated technique has proven to be effective in detecting patient or treatment plan mix-ups with near-perfect accuracy.

## Highlights

• Geometry of catheter implant uniquely identifies patients in breast brachytherapy.
• Electromagnetic tracking captures implant geometry before fraction during check run.
• Analysis shows up to 99.9% accuracy across 6400 patient-treatment plan comparisons.
• 100% specificity and 97.5% sensitivity ensure reliable patient identification.
• Automated geometry verification prevents mix-ups and protects privacy.

## Introduction

Patient safety is alongside treatment success one of the two fundamental principles of health care in general and is also self-evident in radiation therapy. Despite the ever-improving quality of treatments due to technological advances, better teaching, increased error awareness and the implementation of a positive culture of failure, errors in radiotherapy still occur as several reports on critical incidents have shown [1]. Performing high dose rate brachytherapy can be particularly challenging from a risk analysis standpoint as it comprises several risk factors: complex and largely manual procedures, comparatively high single radiation doses and steep dose gradients, the involvement of several different groups of medical staff, only limited time for treatment planning, and a very short period of time between treatment planning and treatment delivery [2]. According to some of the largest databases on critical incidents (Safety in Radiation Oncology database by the International Atomic Energy Agency [3], [4], [5], International Commission on Radiological Protection database [6], and United States Nuclear Regulatory Commission database [7], Radiation oncology incident learning system AAPM/ASTRO [8], [9]) as well as the German Critical Incident Reporting System by the Federal Office for Radiation Protection [10], [11], [12], [13], [14], human errors at least partly contribute to most of the reported events, and to almost all of the very critical events [15], [16], [17], [18], [19]. Alarmingly, Richardson et al. point out that many of the reported events are not related to modern technology, but instead it is a matter of historically frequent and therefore well-known errors like wrong patient or treatment plan, wrong treatment site or incorrect indexer lengths [16].

Among these, the mix-up of patients or treatment plans is one of the simplest errors, but at the same time one of the most serious with a high potential to harm the patient. In external beam radiotherapy, patient mix-ups are less likely as cone-beam computed tomography (CBCT) imaging, surface scanning and dedicated systems for patient identification (biometric or radio frequency identification) are commonly used. In brachytherapy, in contrast, such systems are rarely used which opens the door for mix-ups not being detected. The only technical measure to detect a potential misapplication is the mandatory check cable run before the actual source cable run for irradiation. The check cable is a second cable with a non-radioactive, dummy source that is independent from the source cable and is driven from the afterloader, through the transfer tubes, and into the patient's applicators to simulate the path of the actual radioactive source. While intended to test the catheters for obstructions, it will also indicate if a wrong treatment plan with more catheters than the correct plan is selected since the channel(s) of the surplus catheter(s) are not connected and consequently yield a warning. However, the opposite scenario of a wrong treatment plan with less catheters than the correct plan will remain undetected since the check cable can pass through all implanted catheters, yet does not notice that additional catheters are connected.

In this study, we propose electromagnetic tracking (EMT) as an innovative tool to close the described gap for multi-catheter interstitial breast brachytherapy. In recent years, EMT has gained increasing interest in the field of interstitial brachytherapy (iBT) offering an automated solution for advanced patient-specific quality assurance and pre-treatment verification [20], [21], [22]. The fundamental idea behind the use of EMT in iBT is to assess the geometry of the iBT implant on a fraction basis, in treatment position and without any additional dose exposure to the patient. EMT has been proven capable of detecting common iBT treatment errors like shifts and swaps of catheters [23], [24], [25], incorrect indexer lengths [23], [24], [25], and reconstruction errors [26], [27], [28]. Besides that, it can also detect more subtle interfractional changes of the patient's anatomy [29], [30], [31] or guide the placement of interstitial needles [32], [33]. Here, we assess the possibility to identify patients according to the geometry of their individual catheter implant with the goal of preventing patient or treatment plan mix-ups.

## Materials & methods

### EMT data acquisition and patient cohort

We performed EMT measurements using a prototype afterloader (Flexitron, Elekta Brachytherapy, Veenendaal, The Netherlands) whose check cable has been fitted with an EMT sensor (Aurora, NDI, Waterloo, Canada). The EMT-enabled afterloader provides the same possibilities to define dwell positions and dwell times for the EMT sensor as a clinical afterloader does for the radiation source. To acquire the 3D geometry of the catheter implant, a so-called field generator emitting a heterogeneous magnetic field of roughly 50 × 50 × 50 cm^3^ is placed above the patient and the sensor is automatically moved through all catheters sequentially while constantly recording positional data at a rate of 40 Hz.

In this patient study conducted at the Universitätsklinikum Erlangen (ethics approval number NDI-HDR-1355-14B, 2014, renewed 2020; informed consent was obtained prior to participation), sensor dwell positions were spaced 1 cm apart, the dwell time was set to 1 s, and the sensor speed was 50 cm/s. Measurements were performed after computed tomography (CT) simulation and each treatment fraction, and in treatment position, i.e., without moving the patient between treatment and measurement. For further information about the measurement protocol [30], [34], the technical details of the EMT-enabled afterloader [34], [35], [36] and its accuracy, the reader is referred to previous studies [29], [36], [37], [38] and reviews [20], [21], [39], [40].

The patient cohort comprised patients with carcinoma of the breast who had undergone high dose rate interstitial brachytherapy, either as sole accelerated partial breast irradiation (APBI) or as an adjuvant boost after whole breast irradiation. CT-based implant reconstructions were performed manually by experienced physicists adhering to GEC-ESTRO guidelines. The institutional standard of the CT scan parameters envisaged a slice thickness of 2 mm and an isotropic in-plane pixel size between 0.5 and 0.8 mm (Somatom Sensation or Somatom Go.Open Pro; Siemens Healthineers, Erlangen, Germany).

### Processing of clinical and EMT implant reconstructions

All data processing steps were performed by a custom-developed MATLAB routine (version R2019b, The MathWorks Inc., Massachusetts, USA). The EMT raw data was processed to extract only the sensor positions when the sensor was at rest at a dwell position and data points of the same dwell position were averaged. The implementation of motion compensation through additional EMT reference sensors on the breast surface was necessary to eliminate breathing motion artefacts [41]. The resulting set of 3D points representing the catheter implant is referred to as EMT implant reconstruction.

The CT-based, clinical reconstruction points extracted from the DICOM RT plan file were interpolated and resampled to match the 1 cm-spacing of EMT sensor dwell positions, allowing to establish a point-to-point correspondence between the clinical and the EMT implant reconstruction.

### Implant comparison and similarity measure

To quantify the similarity of a clinical and an EMT implant reconstruction, they were rigidly registered to map the EMT implant reconstruction into the coordinate system of the clinical reconstruction. After that, the Euclidean distances between each pair of corresponding points were calculated, yielding a distribution of geometric differences. To quantify the similarity between the two, we introduced a distance-to-agreement (DTA) parameter (3–10 mm) and a pass rate. The pass rate is defined as the proportion of corresponding point pairs that are closer to each other than the selected DTA parameter. Based on this, a classification into “same patient” or “different patient” was done in the context of a receiver operating characteristic (ROC).

Across the full cohort of 80 patients, and using up to 12 EMT implant reconstructions per patient (post-surgery in the patient bed, after CT simulation on the CT table, and after up to nine treatment fractions), more than 57,000 pairwise comparisons between clinical and EMT implant reconstructions were generated. To simulate the clinical error scenario where a treatment plan is accidentally loaded that contains fewer catheters than the actual implant, only the number of catheters in the treatment plan was considered in the implant comparison.

## Results

**Fig. 1:**
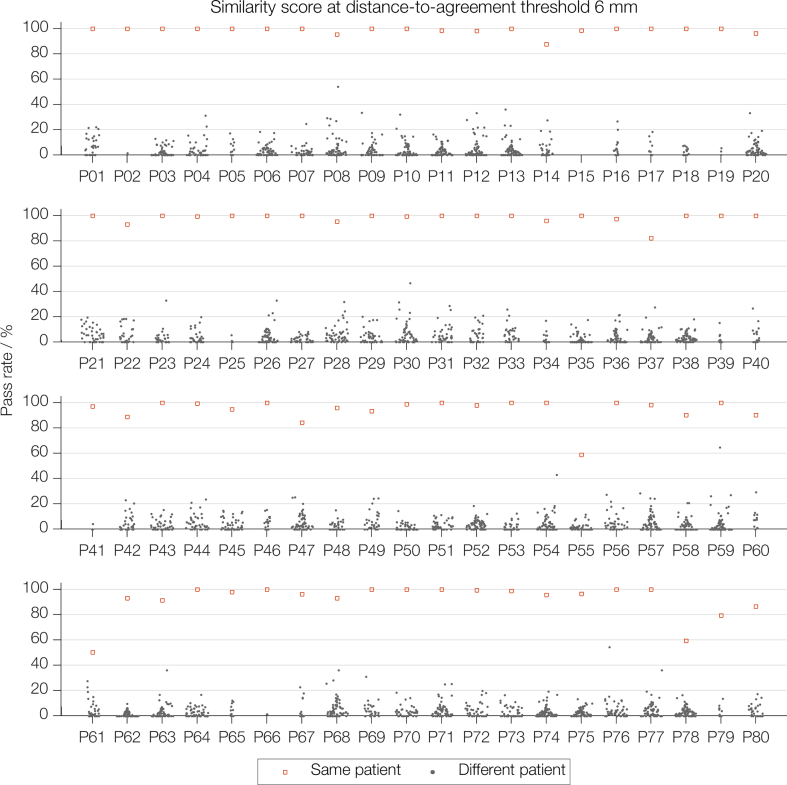
Similarity of the clinical and the EMT implant reconstructions at the first treatment fraction across the entire patient cohort indicated by the pass rate after applying a 6 mm DTA threshold. The pass rate represents the percentage of points in the EMT implant reconstruction that lay closer than the specified DTA from their corresponding points in the clinical reconstruction.

In all cases, the same-patient similarity score, i.e., the pass rate, was highest for all distance thresholds considered (Fig. 1; see also Figures S.1-7). While most of the same-patient similarity scores were above 90%, the majority of different-patient scores were below 40%. This clear margin between the two categories was only interrupted by three special cases (P55, P61, P78). These patients are characterised by comparatively low same-patient scores of 50–60%, however, the corresponding different-patient scores were even lower.

The range of same-patient scores was 25%–100% (5th to 95th percentile: 51%–100%) for a DTA of 4 mm, 50%–100% (5th to 95th percentile: 81%–100%) for a DTA of 6 mm, 76%–100% (5th to 95th percentile: 92%–100%) for a DTA of 8 mm, 85%–100% (5th to 95th percentile: 95%–100%) for a DTA of 10 mm. The highest different-patient score for each threshold was 33% (P08, 4 mm DTA), 65% (P59, 6 mm DTA), 91% (P59, 8 mm DTA), and 95% (P59, 10 mm DTA). The closest agreement between same-patient score and different-patient score was found for P61 (25% vs. 5%), P61 (50% vs. 27%), P59 (100% vs. 91%), and P59 (100% vs. 95%) for thresholds of 4 mm, 6 mm, 8 mm, and 10 mm, respectively.

**Fig. 2:**
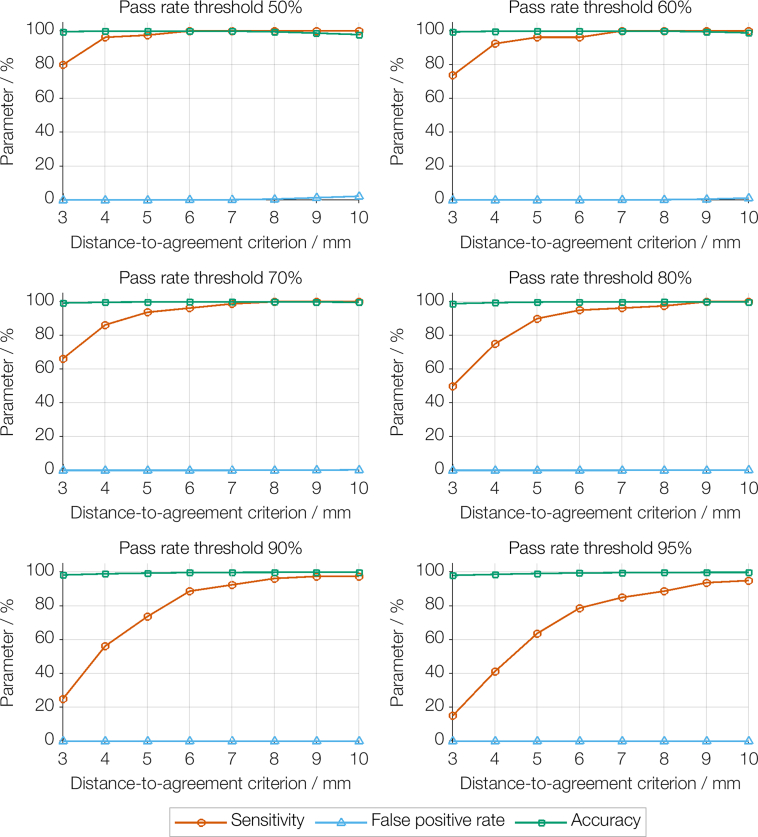
Dependence of important standard statistical parameters of a receiver operating characteristic (sensitivity, false positive rate, and accuracy) on the DTA threshold at different pass rate thresholds.

Within the selected parameter range, the ROC analysis of the classification performance (Fig. 2; Tables 1-6) yielded high values for the accuracy (minimum 97.8%, 99.6% on average) and very low values for the false positive rate (< 2.2%, 0.15% on average), indicating very good specificity, across all pass rate thresholds. Sensitivity showed more variation (range 15–100%) and generally increased with increasing DTA threshold while the gain in sensitivity attenuated towards higher DTA values. At lower pass rate thresholds (50–80%), sensitivity reached a plateau of more than 90% beyond a DTA value of 4–5 mm, whereas at higher pass rate thresholds (90–95%), this was the case only at larger DTA values of 7–9 mm. At lower DTA values (3–4 mm), a substantial drop in sensitivity was noticeable for all pass rate thresholds, with the drop being more pronounced the higher the pass rate threshold was. This suggests that too stringent spatial tolerances, i.e., DTA values, slightly reduce the method's performance.

**Fig. 3:**
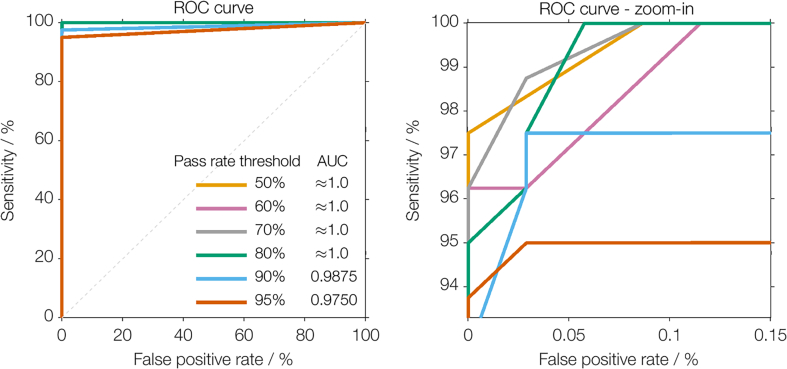
ROC curves and area under the curve (AUC) for different pass rate thresholds (left) with zoom-in of the same plot (right). The diagonal line (dotted) indicates the line of no discrimination, i.e., random classification performance corresponding to an AUC of 0.5.

The classifier exhibited excellent discriminatory power across all tested DTA thresholds (Fig. 3). The areas under the curve (AUC) ranged from 0.9750 to a numerical value of 1, with the actual values marginally below 1. This high performance remained stable over the course of the treatment, i.e., up to five days, with no notable changes observed in the ROC analysis.

## Discussion

The present study was designed to determine whether breast cancer patients treated with multi-catheter interstitial brachytherapy could be identified solely by the geometry of their catheter implant. Several reports on critical incidents have pointed out that patient or treatment plan mix-ups continue to occur in everyday clinical practice, even in modern radio- and brachytherapy [2], [16], [17], [19], [42]. However, absolute numbers on the frequency of errors are scarce and, if they are reported, they aggregate brachytherapy and external beam radiotherapy. According to the German Federal Office for Radiation Protection, 74 instances of patient mix-ups and 87 instances of treatment plan mix-ups were reported as critical incidents in radiotherapy in Germany between 2019 and 2023 [10], [11], [12], [13], [14]. This is despite the fact that mix-ups are fairly easy to detect and avoid, but can have severe consequences.

In contrast to organisational (checklists, standard operating procedures, timeouts) and educational means (continuous training, raising awareness), technical solutions promise to meaningfully complement them as they largely eradicate the aspect of human failure and thus the treatment process can become more fault-tolerant [43], [44], [45]. Hendee and Herman provided expert guidance from a multi-society safety initiative in radiation oncology naming automation and computerization among the most effective and quickly implementable actions to reduce errors, especially compared to training, education and checklists [44]. In a survey on the usability and usefulness of patient identification systems in radiation oncology, Baehr et al. reported that 51% of the participating centres that only used organisational methods to identify patients, i.e., asking for patient-specific identifiers like name and date of birth, experienced one or more near-events of misidentification within the past five years. In centres that used additional technical solutions for identification, the rate was 28% and therefore nearly half as high [46].

The range of such techniques is broad [47] and includes essentially barcode or RFID scanning as well as biometric recognition based on unchanging physical features (palm vein [48] and fingerprint scanning [49], [50], facial recognition [51], [52] and surface imaging [53]). However, due to their permanent nature, biometric information is considered sensitive personal information requiring special protection from misuse for other than medical purposes. The use of non-permanent features is therefore preferential as data protection concerns are circumvented; instead, they might lead to less reliable identification during the treatment course. We hypothesised that the geometry of an iBT implant, i.e., the 3D arrangement of catheters implanted in patients, is unique and could serve as a characteristic feature for identifying patients. So far, capitalising on this idea has been impractical to impossible since fraction-wise pre-treatment implant assessment requires in-room imaging capabilities and substantial time of trained personnel to perform the manual image-based reconstruction.

In this regard, EMT technology offers unprecedented possibilities as it does neither require a direct line-of-sight or ionising radiation, nor any user interaction at all to assess the geometry prior to every treatment fraction. The added value of this information has been demonstrated by numerous studies in recent years, including accurate automatic applicator reconstruction [25], [26], [27], [28], improved needle navigation and placement [33], as well as advanced pre-treatment verification (detection of errors in applicator reconstruction and treatment delivery [23], [24], [25], detection of interfractional changes [29], [30], [54]). In the presented use case of patient identification, the implant geometry is measured prior to treatment, while the implant is checked for obstructions with the check cable. The geometry of the patient to be treated will then be compared to the reference geometry from the treatment plan to be administered. In the event of a discrepancy, i.e., if the measured geometry does not pass the comparison, the user will receive a warning or interlock and will be prompted to reassure that the correct treatment plan has been loaded.

In the current study, we compared the EMT-assessed implant geometry with the implant geometry that is the basis for the clinically administered treatment plan. The first noteworthy result is that the same-patient similarity score was always and by far the highest (see Fig. 1): For an exemplary DTA threshold of 6 mm, 72 out of 80 (90%) patients achieved a pass rate of more than 90%, 76 out of 80 (95%) yielded a pass rate of more than 80%. Only three patients (P55, P61, and P78) deviated notably with pass rates as low as 50–60%. All of these patients had relatively large implants with 17, 16, and 22 catheters, respectively, and showed signs of tissue swelling and potential seroma formation. We suspect these anatomical changes as the cause for the comparatively low pass rates.

These results are in agreement with previously reported geometric deviations for iBT breast implants: In a cohort of 41 patients, Kallis et al. found median deviations of 2.2 mm with the 95th percentile reaching up to 6.5 mm [30]. Interestingly, similar values of 1.7 mm for the median and 6–8 mm for the 95th percentile were found in CT-based prostate iBT reported by Androulakis et al. [29]. In contrast to that, the pass rates for different patients generally did not exceed 40%. The highest different-patient similarity score was 65% for P59, but the same-patient similarity score for this patient reached 100%, leaving a clear margin to distinguish. The consistently high pass rates for intra-patient comparisons, coupled with the clear separation between intra- and inter-patient similarity scores supports the idea that the implant geometry can be used as a patient identifier and demonstrates that the DTA metric is sensitive enough to capture these patient-specific features.

Within the context of a ROC analysis, the dependence of sensitivity and accuracy on the DTA criterion reflects the expected trade-off between spatial accuracy and classification robustness. Increasing the DTA threshold improves sensitivity by relaxing spatial constraints, thereby enhancing the method's ability to capture true intra-patient correspondences. Importantly, the consistently low false positive rate across all tested conditions indicates that this gain in sensitivity did not come at the cost of reduced specificity. This is also reflected in the very high AUC, which approaches 1 and shows no evident dependence on the tested pass rate thresholds. The almost perfect accuracy observed beyond a 6 mm DTA criterion suggests that the applied classifier reliably performs within clinically acceptable tolerances. However, the loss of sensitivity at lower thresholds emphasises the limitations of overly strict DTA criteria, which may exclude valid agreements due to small anatomic changes as well as registration and other uncertainties.

From a clinical quality assurance perspective, the primary objective is to minimise the false positive rate to zero, ensuring that no patient is mixed-up and incorrectly treated, even while aiming for the highest possible sensitivity and accuracy. Conversely, a non-zero false negative rate is acceptable to a certain degree: misclassifying the right patient as wrong only notifies the medical personnel of an alleged discrepancy, prompting them to recheck the selected treatment plan or patient. This second check imposes only minor additional effort relative to the substantial benefit of preventing a potential treatment error. Our findings therefore support the use of moderate DTA criteria (approximately 5–8 mm) and pass rate thresholds (50–80%) as a balanced and clinically meaningful choice for the evaluation of similarity between an implant geometry assessed by EMT pre-treatment and the reference geometry obtained from the CT during treatment planning. It is important to note that this choice of parameters and the underlying evaluation do not take into account the error scenario in which a treatment plan with a larger number of catheters is applied incorrectly. This limitation stems from the fact that such a scenario is unrealistic in clinical routine, as the check cable would detect it when trying to access a channel or catheter that is not connected.

Interstitial implants in breast brachytherapy are virtually unique, so determining their geometry prior to each fraction is a viable option for patient identification. The EMT-based automated technique has proven to be effective in detecting patient or treatment plan mix-ups. When implemented into the mandatory check cable run, it enhances patient safety without compromising workflow efficiency. This demonstrates another beneficial use case for EMT in brachytherapy, alongside accurate applicator reconstruction, improved needle navigation and placement, and advanced pre-treatment verification.

## CRediT authorship contribution statement

**Christopher Dürrbeck:** Writing – review & editing, Writing – original draft, Visualization, Validation, Software, Resources, Project administration, Methodology, Investigation, Formal analysis, Data curation, Conceptualization. **Vratislav Strnad:** Writing – review & editing. **Christoph Bert:** Writing – review & editing, Supervision, Resources, Project administration, Funding acquisition, Conceptualization.

## Declaration of competing interest

The authors declare the following financial interests/personal relationships which may be considered as potential competing interests:

This project was partially funded by an unrestricted research grant from Elekta Brachytherapy.
