# Supplementary material for: Implant geometry as a patient-specific identifier in breast brachytherapy: leveraging electromagnetic tracking to prevent treatment mix-ups
Source: Tech Innov Patient Support Radiat Oncol. 2026 May 22;39:100415. doi: 10.1016/j.tipsro.2026.100415 (PMC13321395; doi:10.1016/j.tipsro.2026.100415)
Supplement: Supplementary file 1 — Supplementary material [file mmc1.docx]

Implant geometry as a patient-specific identifier in breast brachytherapy: leveraging electromagnetic tracking to prevent treatment mix-ups

Christopher Dürrbeck ^a,b^, Vratislav Strnad ^a,b^, Christoph Bert ^a,b^

^a^ Department of Radiation Oncology, Universitätsklinikum Erlangen, Friedrich-Alexander-Universität Erlangen-Nürnberg, Erlangen

^b^ Comprehensive Cancer Center, Universitätsklinikum Erlangen, Erlangen

**Corresponding author**

Christopher Dürrbeck

Department of Radiation Oncology

Universitätsklinikum Erlangen

Universitätsstraße 27

e-mail: [christopher.duerrbeck@fau.de](mailto:christopher.duerrbeck@fau.de)

phone: +49 9131 8544920

Keywords: patient identification, treatment error, electromagnetic tracking, interstitial brachytherapy, quality assurance, error detection, breast cancer

Short title: Patient identification in breast iBT using EMT

Highlights:

- Geometry of catheter implant uniquely identifies patients in breast brachytherapy
- Electromagnetic tracking captures implant geometry before fraction during check run
- Analysis shows up to 99.9% accuracy across 6400 patient-treatment plan comparisons
- 100% specificity and 97.5% sensitivity ensure reliable patient identification
- Automated geometry verification prevents mix-ups and protects privacy

Word count, manuscript: 2850

Word count, abstract: 245

# Supplementary Data

## Figures


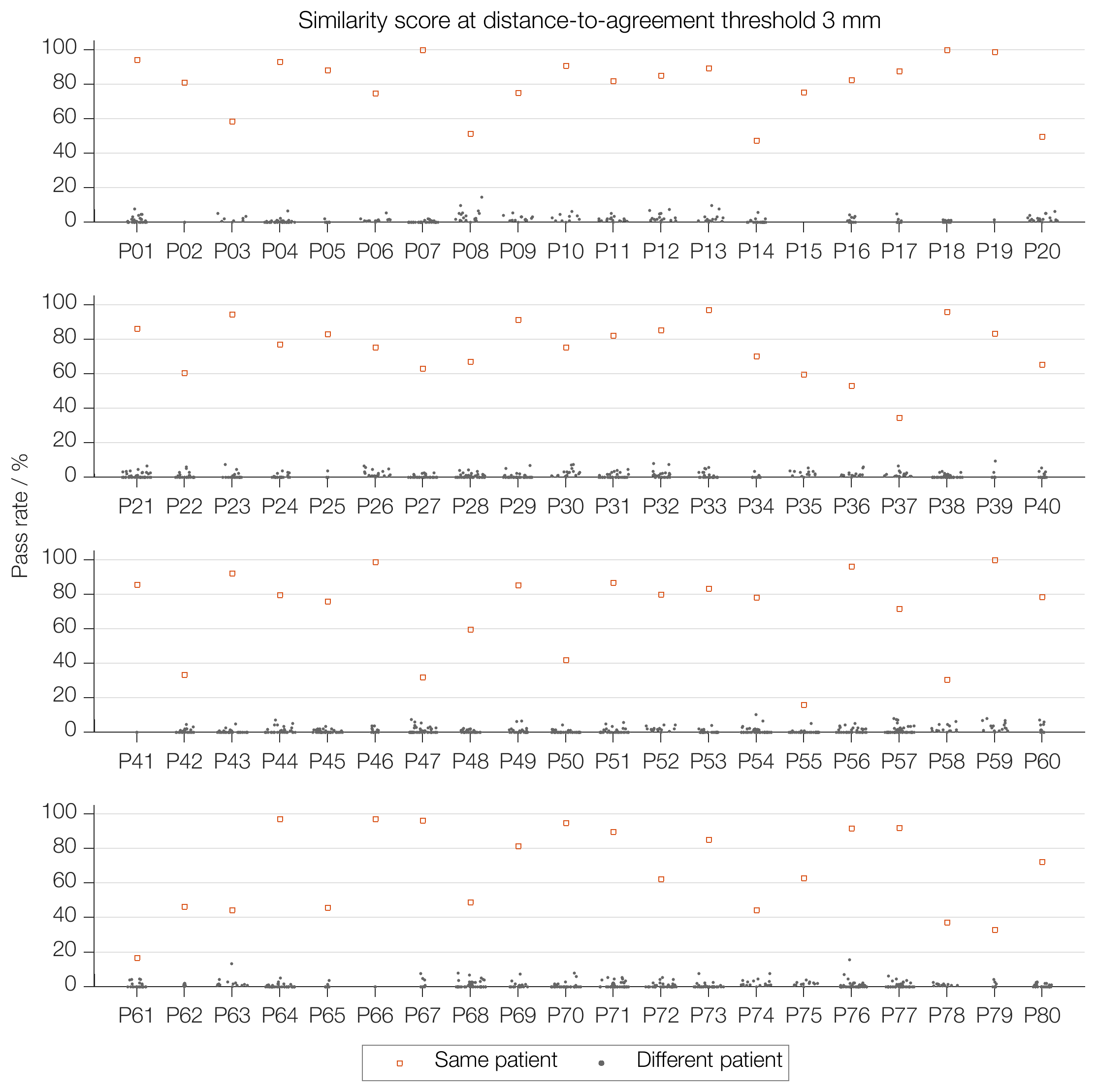


Figure S. 1 Similarity of the clinical and the EMT implant reconstructions at the first treatment fraction across the entire patient cohort indicated by the pass rate after applying a 3 mm DTA threshold. The pass rate represents the percentage of points in the EMT implant reconstruction that did not lie further away from their corresponding points in the clinical reconstruction than the specified DTA.


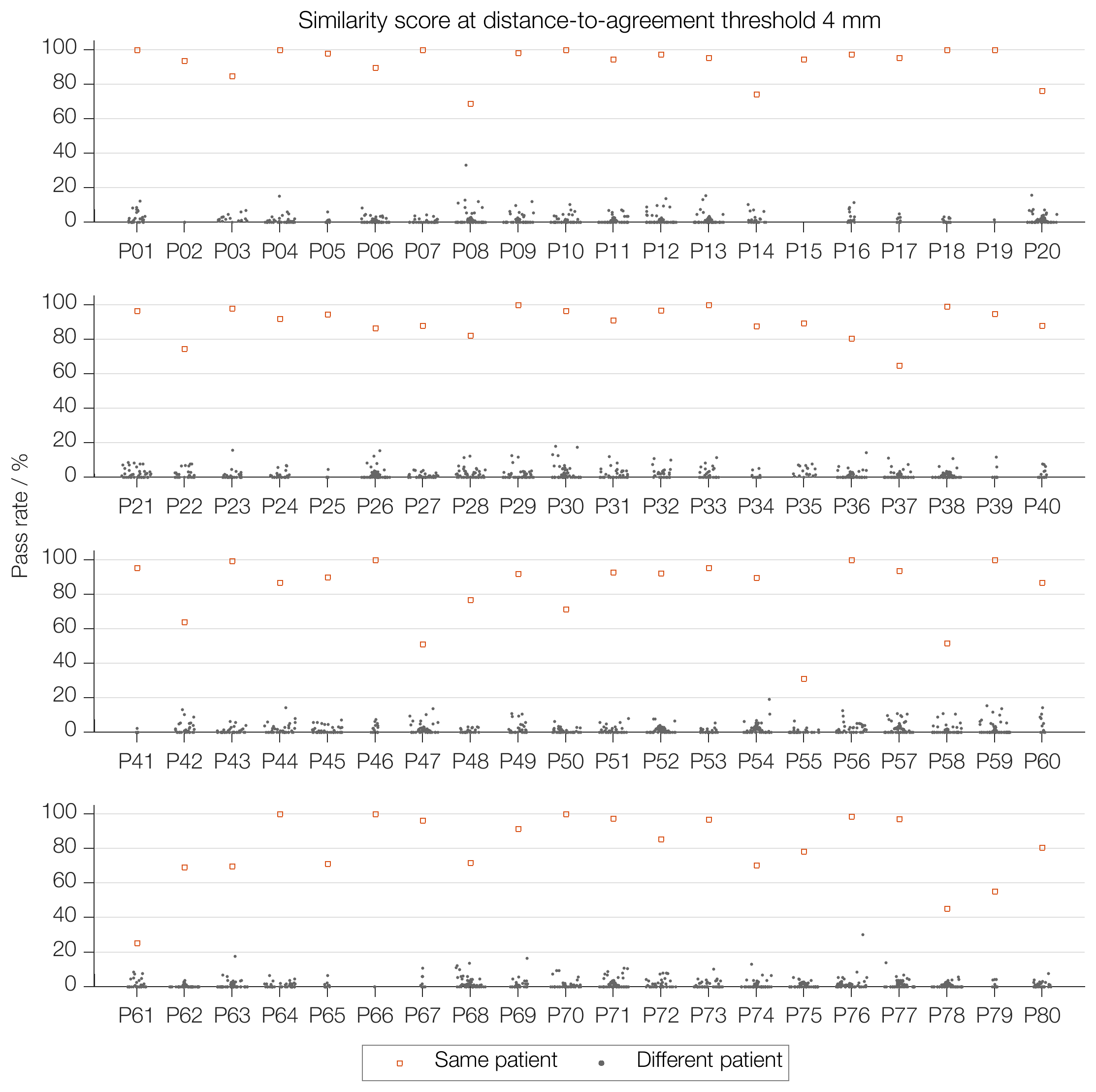


Figure S. 2 Similarity of the clinical and the EMT implant reconstructions at the first treatment fraction across the entire patient cohort indicated by the pass rate after applying a 4 mm DTA threshold. The pass rate represents the percentage of points in the EMT implant reconstruction that did not lie further away from their corresponding points in the clinical reconstruction than the specified DTA.


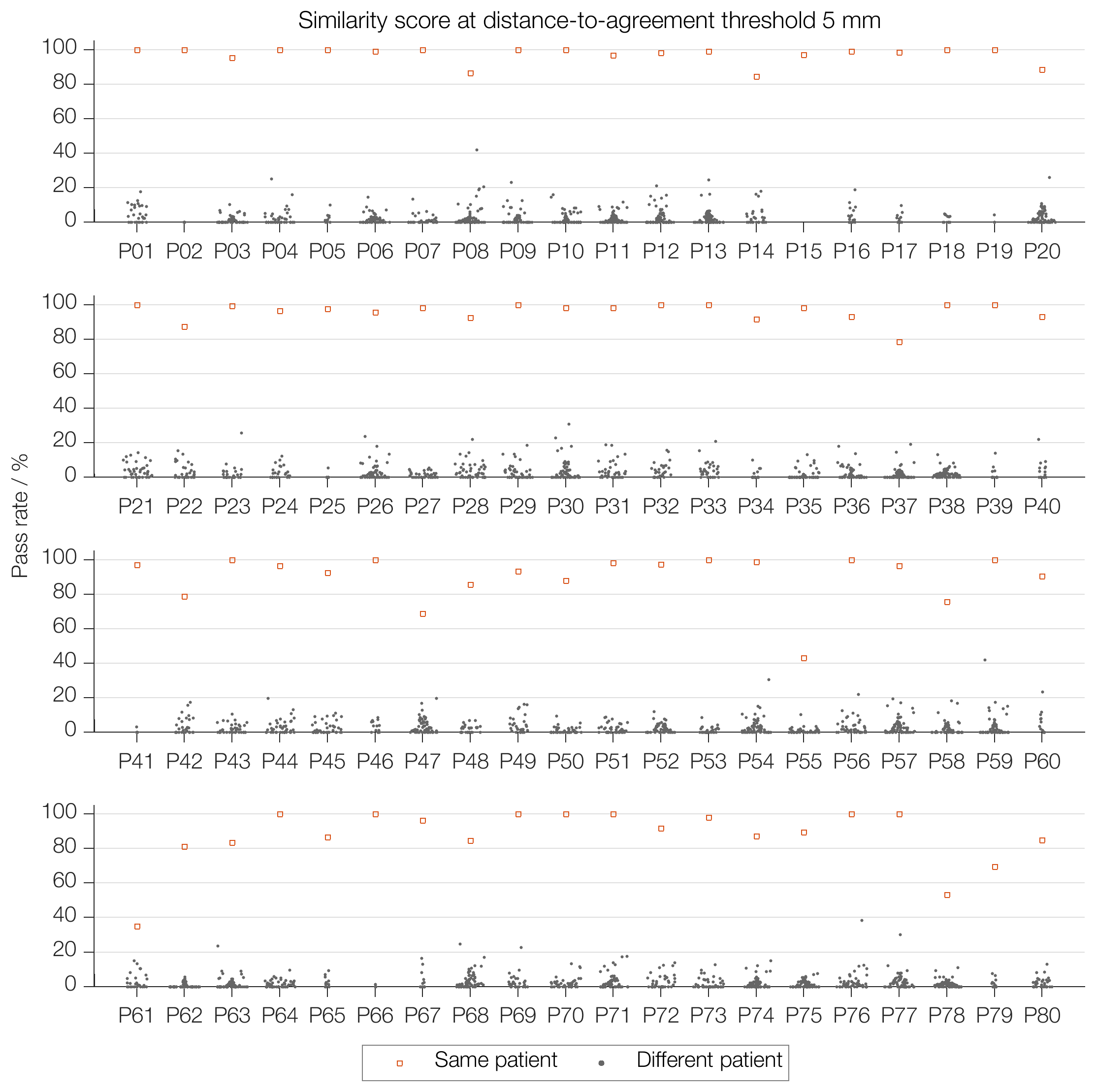


Figure S. 3 Similarity of the clinical and the EMT implant reconstructions at the first treatment fraction across the entire patient cohort indicated by the pass rate after applying a 5 mm DTA threshold. The pass rate represents the percentage of points in the EMT implant reconstruction that did not lie further away from their corresponding points in the clinical reconstruction than the specified DTA.


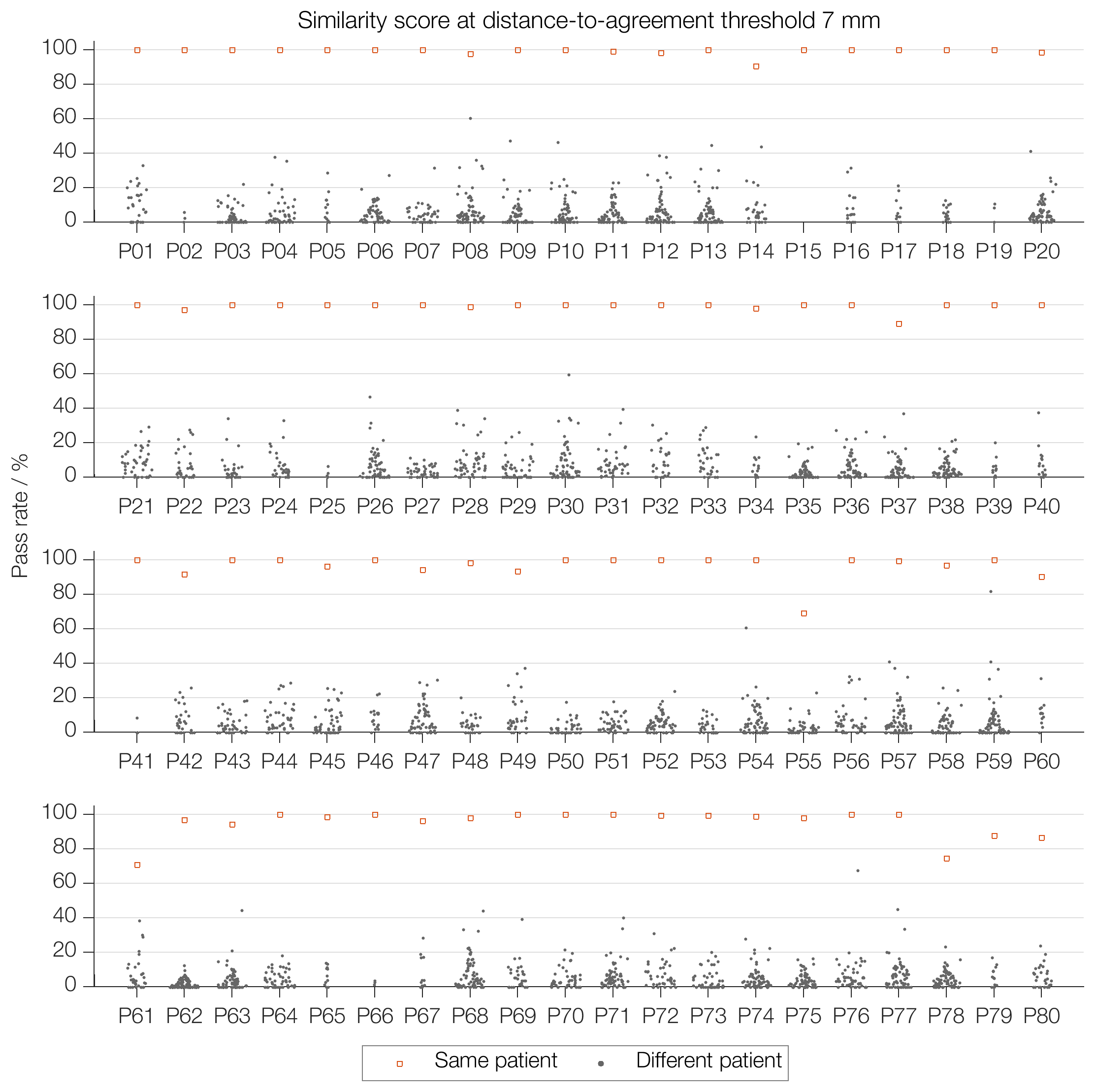


Figure S. 4 Similarity of the clinical and the EMT implant reconstructions at the first treatment fraction across the entire patient cohort indicated by the pass rate after applying a 7 mm DTA threshold. The pass rate represents the percentage of points in the EMT implant reconstruction that did not lie further away from their corresponding points in the clinical reconstruction than the specified DTA.


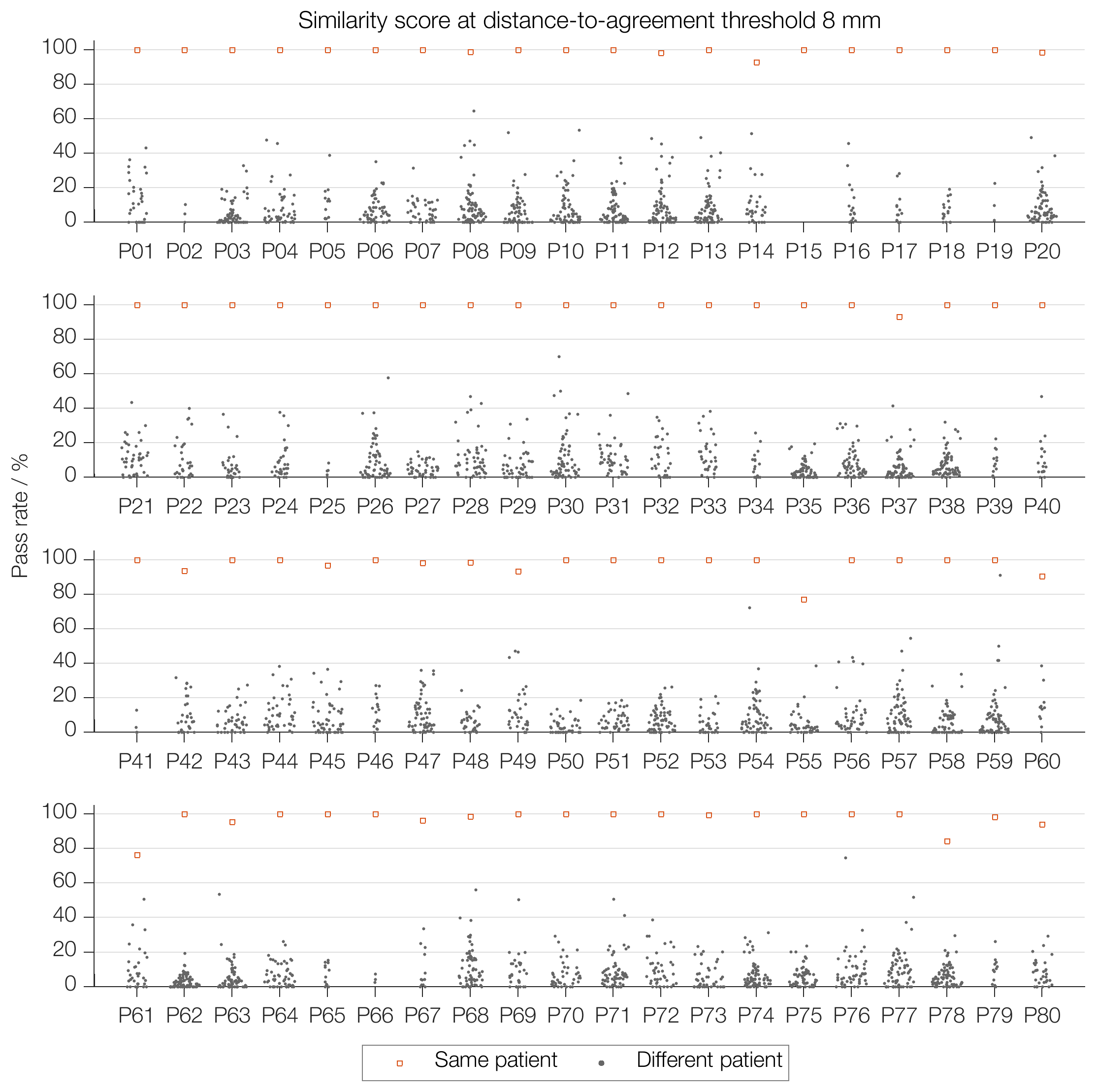


Figure S. 5 Similarity of the clinical and the EMT implant reconstructions at the first treatment fraction across the entire patient cohort indicated by the pass rate after applying a 8 mm DTA threshold. The pass rate represents the percentage of points in the EMT implant reconstruction that did not lie further away from their corresponding points in the clinical reconstruction than the specified DTA.


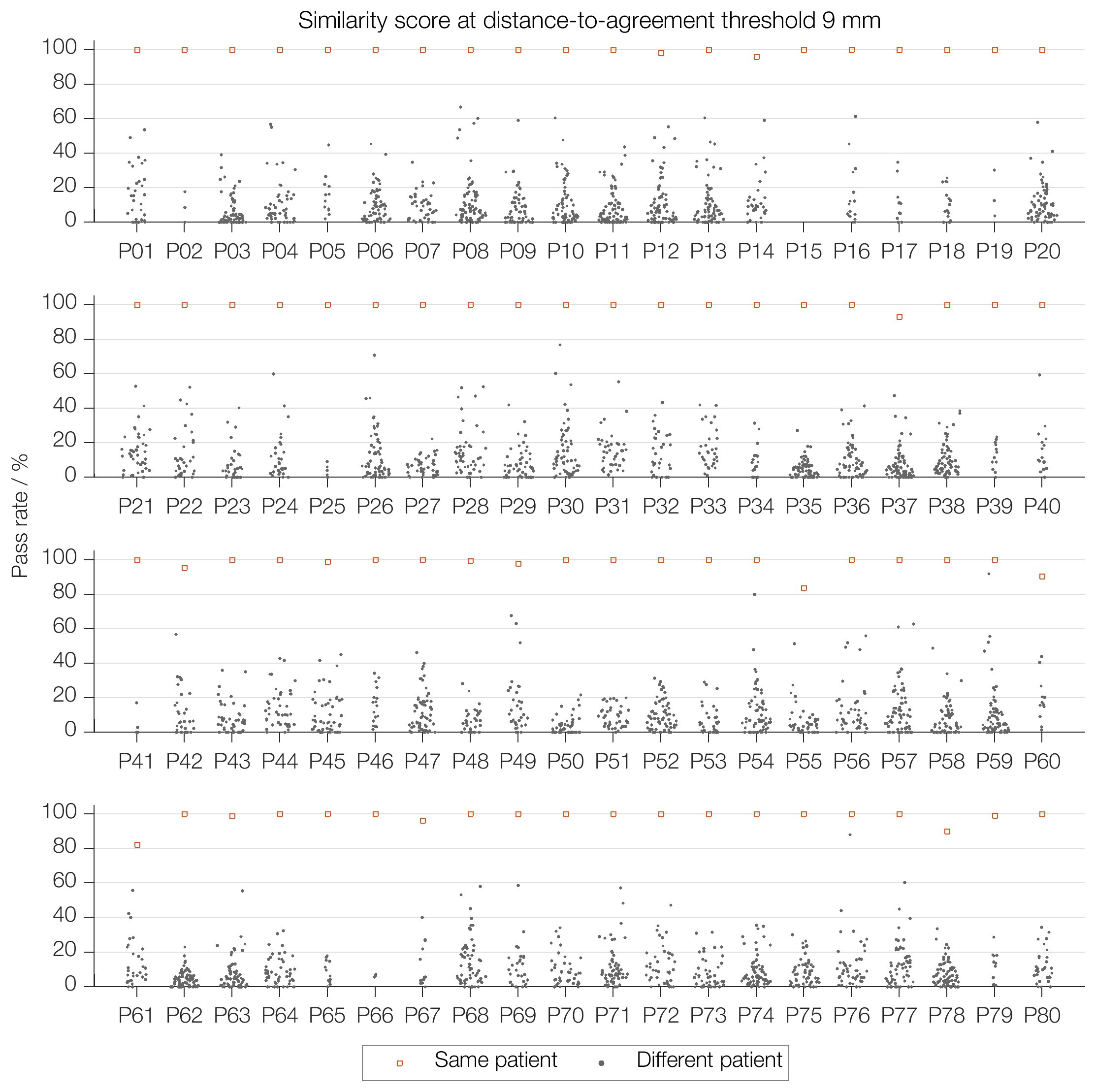


Figure S. 6 Similarity of the clinical and the EMT implant reconstructions at the first treatment fraction across the entire patient cohort indicated by the pass rate after applying a 9 mm DTA threshold. The pass rate represents the percentage of points in the EMT implant reconstruction that did not lie further away from their corresponding points in the clinical reconstruction than the specified DTA.


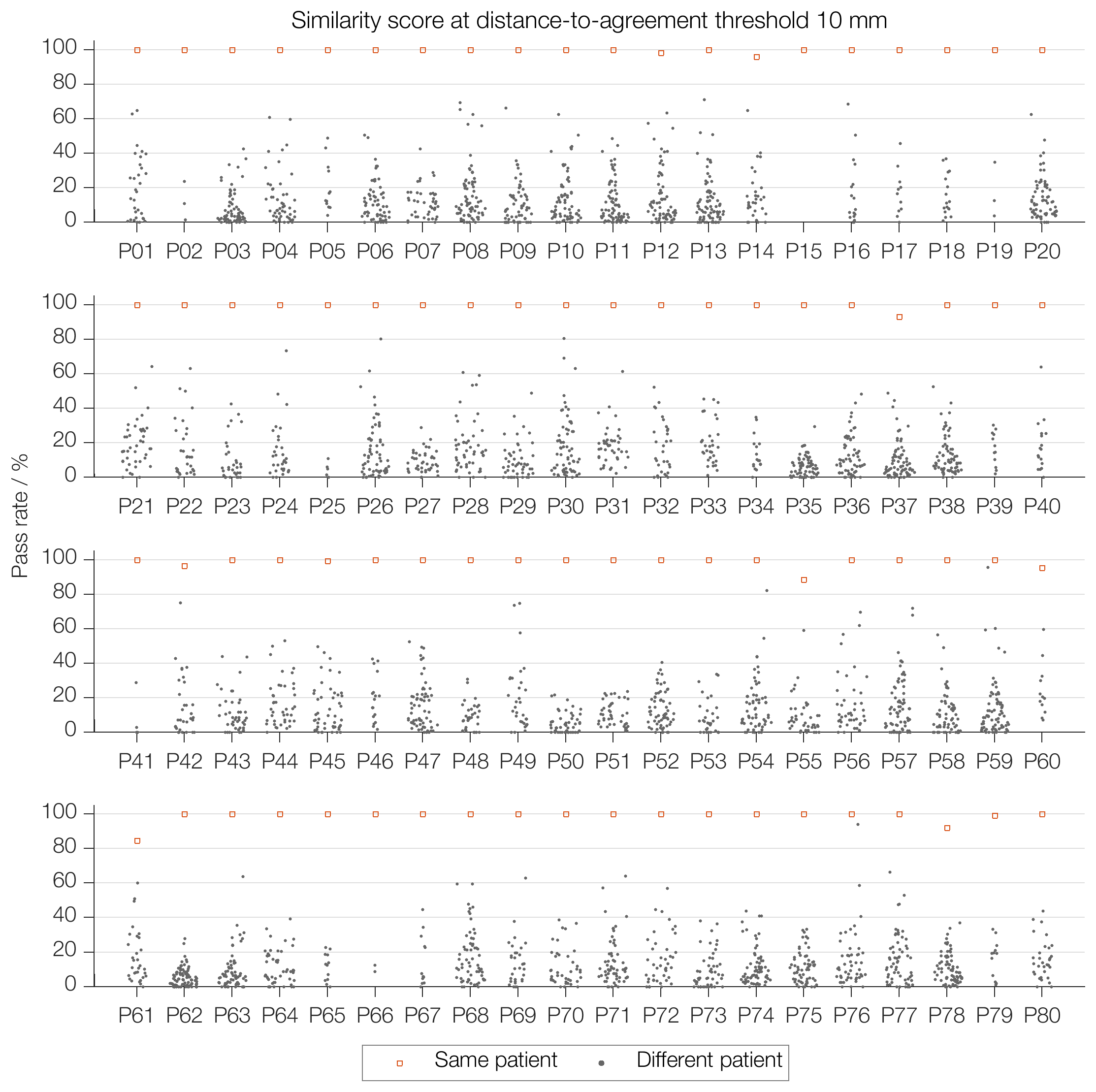


Figure S. 7 Similarity of the clinical and the EMT implant reconstructions at the first treatment fraction across the entire patient cohort indicated by the pass rate after applying a 10 mm DTA threshold. The pass rate represents the percentage of points in the EMT implant reconstruction that did not lie further away from their corresponding points in the clinical reconstruction than the specified DTA.

## Tables

## Table 1. Pass rate threshold 50%

| Parameter / DTA threshold | 3 mm | 4 mm | 5 mm | 6 mm | 7 mm | 8 mm | 9 mm | 10 mm |
| --- | --- | --- | --- | --- | --- | --- | --- | --- |
| Sensitivity | 80.00% | 96.25% | 97.50% | 100.00% | 100.00% | 100.00% | 100.00% | 100.00% |
| Specificity | 100.00% | 100.00% | 100.00% | 99.91% | 99.86% | 99.48% | 98.68% | 97.78% |
| False positive rate | 0.00% | 0.00% | 0.00% | 0.09% | 0.14% | 0.52% | 1.32% | 2.22% |
| False negative rate | 20.00% | 3.75% | 2.50% | 0.00% | 0.00% | 0.00% | 0.00% | 0.00% |
| Precision | 100.00% | 100.00% | 100.00% | 96.39% | 94.12% | 81.63% | 63.49% | 50.96% |
| Accuracy | 99.55% | 99.92% | 99.94% | 99.92% | 99.86% | 99.49% | 98.71% | 97.83% |

## Table 2. Pass rate threshold 60%

| Parameter / DTA threshold | 3 mm | 4 mm | 5 mm | 6 mm | 7 mm | 8 mm | 9 mm | 10 mm |
| --- | --- | --- | --- | --- | --- | --- | --- | --- |
| Sensitivity | 73.75% | 92.50% | 96.25% | 96.25% | 100.00% | 100.00% | 100.00% | 100.00% |
| Specificity | 100.00% | 100.00% | 100.00% | 99.97% | 99.88% | 99.86% | 99.51% | 98.85% |
| False positive rate | 0.00% | 0.00% | 0.00% | 0.03% | 0.12% | 0.14% | 0.49% | 1.15% |
| False negative rate | 26.25% | 7.50% | 3.75% | 3.75% | 0.00% | 0.00% | 0.00% | 0.00% |
| Precision | 100.00% | 100.00% | 100.00% | 98.72% | 95.24% | 94.12% | 82.47% | 66.67% |
| Accuracy | 99.41% | 99.83% | 99.92% | 99.89% | 99.89% | 99.86% | 99.52% | 98.87% |

## Table 3. Pass rate threshold 70%

| Parameter / DTA threshold | 3 mm | 4 mm | 5 mm | 6 mm | 7 mm | 8 mm | 9 mm | 10 mm |
| --- | --- | --- | --- | --- | --- | --- | --- | --- |
| Sensitivity | 66.25% | 86.25% | 93.75% | 96.25% | 98.75% | 100.00% | 100.00% | 100.00% |
| Specificity | 100.00% | 100.00% | 100.00% | 100.00% | 99.97% | 99.91% | 99.86% | 99.68% |
| False positive rate | 0.00% | 0.00% | 0.00% | 0.00% | 0.03% | 0.09% | 0.14% | 0.32% |
| False negative rate | 33.75% | 13.75% | 6.25% | 3.75% | 1.25% | 0.00% | 0.00% | 0.00% |
| Precision | 100.00% | 100.00% | 100.00% | 100.00% | 98.75% | 96.39% | 94.12% | 87.91% |
| Accuracy | 99.24% | 99.69% | 99.86% | 99.92% | 99.94% | 99.92% | 99.86% | 99.69% |

## Table 4. Pass rate threshold 80%

| Parameter / DTA threshold | 3 mm | 4 mm | 5 mm | 6 mm | 7 mm | 8 mm | 9 mm | 10 mm |
| --- | --- | --- | --- | --- | --- | --- | --- | --- |
| Sensitivity | 50.00% | 75.00% | 90.00% | 95.00% | 96.25% | 97.50% | 100.00% | 100.00% |
| Specificity | 100.00% | 100.00% | 100.00% | 100.00% | 99.97% | 99.97% | 99.94% | 99.86% |
| False positive rate | 0.00% | 0.00% | 0.00% | 0.00% | 0.03% | 0.03% | 0.06% | 0.14% |
| False negative rate | 50.00% | 25.00% | 10.00% | 5.00% | 3.75% | 2.50% | 0.00% | 0.00% |
| Precision | 100.00% | 100.00% | 100.00% | 100.00% | 98.72% | 98.73% | 97.56% | 94.12% |
| Accuracy | 98.87% | 99.44% | 99.77% | 99.89% | 99.89% | 99.92% | 99.94% | 99.86% |

## Table 5. Pass rate threshold 90%

| Parameter / DTA threshold | 3 mm | 4 mm | 5 mm | 6 mm | 7 mm | 8 mm | 9 mm | 10 mm |
| --- | --- | --- | --- | --- | --- | --- | --- | --- |
| Sensitivity | 25.00% | 56.25% | 73.75% | 88.75% | 92.50% | 96.25% | 97.50% | 97.50% |
| Specificity | 100.00% | 100.00% | 100.00% | 100.00% | 100.00% | 99.97% | 99.97% | 99.94% |
| False positive rate | 0.00% | 0.00% | 0.00% | 0.00% | 0.00% | 0.03% | 0.03% | 0.06% |
| False negative rate | 75.00% | 43.75% | 26.25% | 11.25% | 7.50% | 3.75% | 2.50% | 2.50% |
| Precision | 100.00% | 100.00% | 100.00% | 100.00% | 100.00% | 98.72% | 98.73% | 97.50% |
| Accuracy | 98.31% | 99.02% | 99.41% | 99.75% | 99.83% | 99.89% | 99.92% | 99.89% |

## Table 6. Pass rate threshold 95%

| Parameter / DTA threshold | 3 mm | 4 mm | 5 mm | 6 mm | 7 mm | 8 mm | 9 mm | 10 mm |
| --- | --- | --- | --- | --- | --- | --- | --- | --- |
| Sensitivity | 15.00% | 41.25% | 63.75% | 78.75% | 85.00% | 88.75% | 93.75% | 95.00% |
| Specificity | 100.00% | 100.00% | 100.00% | 100.00% | 100.00% | 100.00% | 100.00% | 99.97% |
| False positive rate | 0.00% | 0.00% | 0.00% | 0.00% | 0.00% | 0.00% | 0.00% | 0.03% |
| False negative rate | 85.00% | 58.75% | 36.25% | 21.25% | 15.00% | 11.25% | 6.25% | 5.00% |
| Precision | 100.00% | 100.00% | 100.00% | 100.00% | 100.00% | 100.00% | 100.00% | 98.70% |
| Accuracy | 98.09% | 98.68% | 99.18% | 99.52% | 99.66% | 99.75% | 99.86% | 99.86% |
